# Clinical response after laparoscopic fenestration of symptomatic hepatic cysts: a systematic review and meta-analysis

**DOI:** 10.1007/s00464-018-6490-8

**Published:** 2018-10-17

**Authors:** Lucas H. P. Bernts, Sebastiaan G. Echternach, Wietske Kievit, Camiel Rosman, Joost P. H. Drenth

**Affiliations:** 1 Department of Gastroenterology and Hepatology, Radboudumc, P.O. Box 9101, 6500 HB Nijmegen, The Netherlands; 2 Department for Health Evidence, Radboudumc, Nijmegen, The Netherlands; 3 Department of Surgery, Radboudumc, Nijmegen, The Netherlands

**Keywords:** Hepatic cysts, Polycystic liver disease, Laparoscopic fenestration, Clinical outcomes

## Abstract

**Background:**

Laparoscopic fenestration is one of the treatment options for symptomatic hepatic cysts, either solitary or in context of polycystic liver disease (PLD), but indications, efficacy and surgical techniques are under debate.

**Methods:**

A systematic literature search (1950–2017) of PubMed, Embase, Web of Science and the Cochrane Library was performed (CRD42017071305). Studies assessing symptomatic relief or symptomatic recurrence after laparoscopic fenestration in patients with symptomatic, non-parasitic, hepatic cysts were included. Complications were scored according to Clavien–Dindo. Methodological quality was assessed by Newcastle–Ottawa scale (NOS) for cohort studies. Pooled estimates were calculated using a random effects model for meta-analysis.

**Results:**

Out of 5277 citations, 62 studies with a total of 1314 patients were included. Median NOS-score was 6 out of 9. Median follow-up duration was 30 months. Symptomatic relief after laparoscopic fenestration was 90.2% (95% CI 84.3–94.9). Symptomatic recurrence was 9.6% (95% CI 6.9–12.8) and reintervention rate was 7.1% (95% CI 5.0–9.4). Post-operative complications occurred in 10.8% (95% CI 8.1–13.9) and major complications in 3.3% (95% CI 2.1–4.7) of patients. Procedure-related mortality was 1.0% (95% CI 0.5–1.6). In a subgroup analysis of PLD patients (*n* = 146), symptomatic recurrence and reintervention rates were significantly higher with respective rates of 33.7% (95% CI 18.7–50.4) and 26.4% (95% CI 12.6–43.0). Complications were more frequent in PLD patients, with a rate of 29.3% (95% CI 16.0–44.5).

**Conclusions:**

Laparoscopic fenestration is an effective procedure for treatment of symptomatic hepatic cysts with a low symptomatic recurrence rate. The symptomatic recurrence rate and risk of complications are significantly higher in PLD patients.

**Electronic supplementary material:**

The online version of this article (10.1007/s00464-018-6490-8) contains supplementary material, which is available to authorized users.

Simple hepatic cysts are fluid-filled cavities that arise from malformations of the ductal plate during embryonic development. Simple hepatic cysts are a relatively common finding as it is estimated to be present in 2.5–18% of the general population [1, 2]. The presence of multiple cysts, arbitrarily > 10, is defined as polycystic liver disease (PLD) [3] and is usually part of the phenotype of two inherited disorders: autosomal dominant polycystic kidney disease (ADPKD) or autosomal dominant polycystic liver disease (ADPLD). Regardless of underlying pathology, these patients are at risk to develop large cysts, arbitrarily defined as > 5 cm in diameter. Large cysts may cause symptoms such as pain, loss of appetite, early satiety, nausea or dyspnea, sometimes causing a considerable decrease in quality of life [3, 4]. As such, treatment of large symptomatic cysts is indicated. Treatment options for large cysts comprise laparoscopic fenestration, also termed laparoscopic deroofing or unroofing, and percutaneous aspiration sclerotherapy [5].

Laparoscopic fenestration combines cyst fluid aspiration, followed by excision of extra-hepatic cyst wall in a single laparoscopic procedure. The surgical approach of large hepatic cysts has gained popularity since the 1990s, especially after the introduction of laparoscopy. As usual in surgical practice, operative treatment has been gradually adopted in routine clinical care without valid comparison. Multiple cohort studies, however, suggest that laparoscopic fenestration is effective and safe in selected populations. Some surgeons routinely apply omentopexy (also termed omentoplasty, omental transposition or greater omentum flap), a procedure that applies omental tissue in the residual cyst cavity to prevent symptomatic recurrence. The merits and risks of omentopexy over and beyond mere laparoscopic fenestration are unexplored.

Percutaneous aspiration sclerotherapy is an alternative approach that percutaneously places a pigtail catheter in the cyst cavity to evacuate hepatic cyst fluid. After complete drainage, a sclerosing agent (e.g. ethanol, tetracycline, polidocanol) is injected in the cyst which destroys the inner epithelial lining resulting in regression of the cyst. A recent clinical guideline suggests that symptomatic simple hepatic cysts may better be managed with laparoscopic fenestration rather than percutaneous aspiration sclerotherapy with the restriction of low quality of evidence [6]. It is imperative to quantify the benefits and risks of laparoscopic fenestration and to grade the evidence on this topic.

The purpose of this study was therefore to assess the efficacy and safety of laparoscopic fenestration using a systematic review of the literature. The primary goal of treatment is alleviation of clinical symptoms, hence our focus on cohort studies and clinical trials that assessed symptomatic relief or symptomatic recurrence. We aim to give a comprehensive summary of reported efficacy and safety rates of laparoscopic fenestration to aid in clinical decision-making when faced with symptomatic hepatic cysts.

## Materials and methods

We conducted a systematic review of studies that evaluated the efficacy of laparoscopic fenestration for symptomatic simple hepatic cysts. This study was reported according to the Preferred Reporting Items for Systematic Reviews and Meta-Analyses (PRISMA) guidelines [7] and the Meta-analysis of Observational Studies in Epidemiology (MOOSE) checklist [8] (Supplementary File 1). The study protocol was registered in the Prospero database of systematic reviews (CRD42017071305) on 10 July 2017.

### Eligibility criteria

We included cohort studies and clinical trials of adult patients with one or more simple (non-parasitic, non-neoplastic) and symptomatic hepatic cysts (excluding choledochal cysts or hepatic foregut cysts), either solitary or in context of PLD, that underwent laparoscopic surgery with minimal resection of healthy liver parenchyma (e.g. fenestration, deroofing, unroofing). We included studies that assessed symptomatic relief and/or symptomatic recurrence. We excluded case reports, overlapping datasets, reviews, unpublished data and conference abstracts. We excluded studies with a mean or median follow-up < 6 months. For practical reasons, only articles in the following languages were included: Dutch, English, French, German, Italian and Spanish.

### Literature search strategy

We systematically searched the electronic databases of PubMed MEDLINE, Embase, Web of Science and the Cochrane Library from inception to 18 July 2017, without any restrictions. The search strategy combined terms related to hepatic cysts and laparoscopic interventions. The search terms were composed in collaboration with an experienced medical librarian. Exact search terms are presented in Supplementary File 2. If no full-text article was available, the original authors were emailed in order to gain access. References of included studies were checked for additional studies missed in the primary search. All identified records were exported to citation management program EndNote X8 (Clarivate Analytics, Philadelphia, PA, USA) for deduplication, which was performed according to a published protocol [9]. After deduplication, all records were exported to the browser-based systematic review management program Covidence (Veritas Health Innovation, Melbourne, Australia. Available at http://www.covidence.org). First, two investigators (LB and SE) independently screened title and abstract to determine the eligibility of each study. Second, the full-text of all included abstracts was independently assessed by the same investigators. Disagreements in both screening phases were resolved through discussion between the two investigators. Any remaining disagreement between reviewers was resolved through discussion with a third reviewer (CR, JD).

### Data extraction

All data were extracted using standardised forms by one investigator (LB). Cases of uncertainty about data extraction were resolved through discussion between two investigators. Original data of four studies were requested by email. One author was able to send the additional data required for inclusion [10]. Data extraction was checked for errors by random sampling of 10% of included studies by a second investigator (SE), which did not show any errors. Our primary outcomes were symptomatic relief (i.e. full or partial symptomatic relief) directly after surgery and symptomatic recurrence (recurrent symptoms with refilling or recurrent symptoms without confirmation of refilling on imaging) during long-term follow-up. Secondary outcomes were study characteristics, patient characteristics, reintervention rates, operative time, hospital stay, conversion to laparotomy and surgical technique. Reported rates of procedure-related complications and mortality were extracted. Reported post-operative complications were scored according to the Clavien–Dindo classification [11] by one investigator (LB). Grade I and II were regarded as minor complications and grade III, IV and V as major complications.

### Risk of bias assessment

We used the Newcastle–Ottawa scale for cohort studies to assess the risk of bias within individual studies. Adaptations were made a priori to make the scale more specific for our research question (Supplementary File 3). Using this scale, studies were scored on selection of study groups, the inclusion of a control group, the comparability of groups and the ascertainment of outcome of interest. Studies were independently scored by two investigators (LB, SE). Disagreements were resolved through discussion between two investigators.

### Data synthesis and analysis

For meta-analysis of reported rates, pooled estimates and 95% confidence intervals (CI) were calculated using a random effects model for meta-analysis of prevalence, using MetaXL 5.2 (EpiGear, Sunrise Beach, Australia. Available at http://www.epigear.com). When comparing means, not overlapping 95% CI were considered significant. When comparing medians, *p* value was calculated with Mann–Whitney test in GraphPad Prism 5 (GraphPad Software, La Jolla, CA, USA), *p* < 0.05 was considered significant.

Heterogeneity for pooled estimates was assessed using the *I*^2^ statistic, which describes the percentage of total variation across studies that is due to heterogeneity rather than chance. As we included a large number of studies, Cochran’s *Q* and *p* values are less practical for assessing heterogeneity [12]. Low, moderate and high heterogeneity was defined as an *I*^2^ value above 25%, 50% or 75%, respectively [12]. All *I*^2^ values were calculated with MetaXL.

Publication bias was assessed by generating funnel plots, where the standard error is plotted against the double arcsine transformed prevalence estimates of individual studies. Likelihood of publication bias was quantified using the Luis Furuya-Kanamori asymmetry index (LFK-index). An LFK-index within 1 or − 1 indicates no asymmetry. An LFK-index exceeding 1 or − 1 but within 2 or − 2 indicates minor asymmetry. An LFK-index exceeding 2 or − 2 indicates major asymmetry [13]. LFK-indices and funnel plots were generated with MetaXL.

### Subgroup analyses

Potential causes of heterogeneity, as such influences on pooled estimates, were investigated by performing pre-specified subgroup analyses of underlying disease, different surgical techniques, study design, publication date and follow-up duration. Subgroups of non-categorical parameters were made by splitting included studies into two groups: 1: equal or below the median and 2: above the median.

All figures were made with Microsoft PowerPoint 2007 (Microsoft Corporation, Redmond, WA, USA) and GraphPad Prism 5.

## Results

### Systematic search

**Fig. 1:**
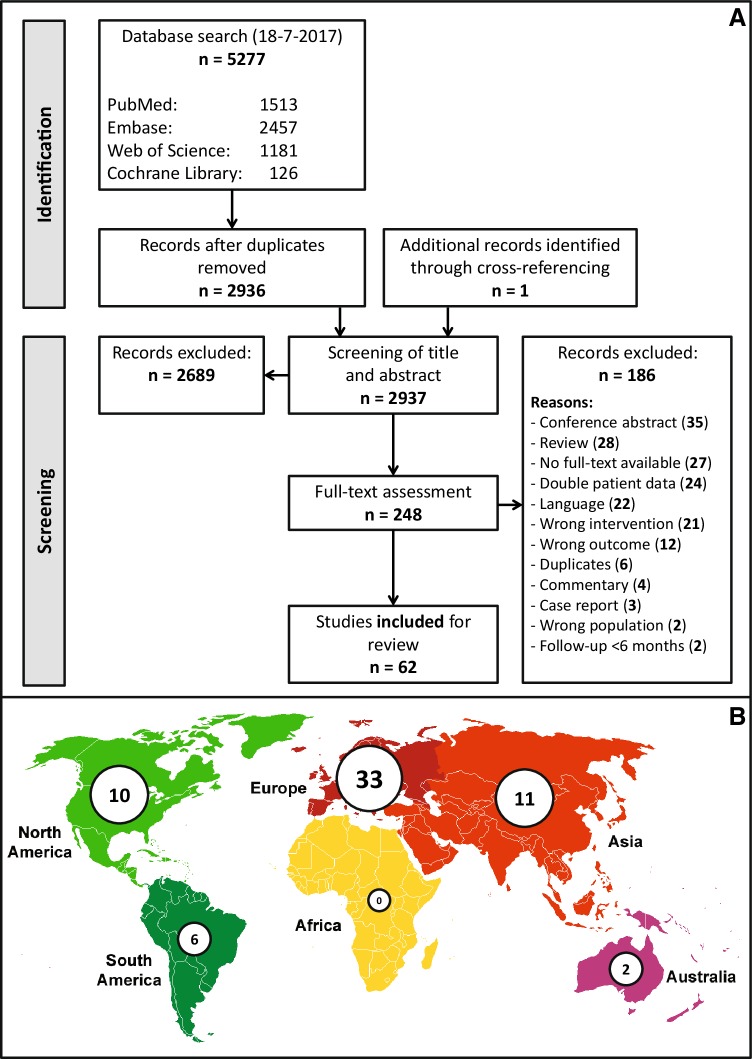
**A** PRISMA diagram. Flow chart representing literature search and elements of systematic review (identification and screening). **B** Illustrative schematic of country of origin of included studies. The number of inclusions per continent is shown

The systematic search identified 5278 citations. Ultimately, 62 studies were included for this systematic review (Fig. 1A). Citations are presented in the supplementary files.

### Study characteristics

**Fig. 2:**
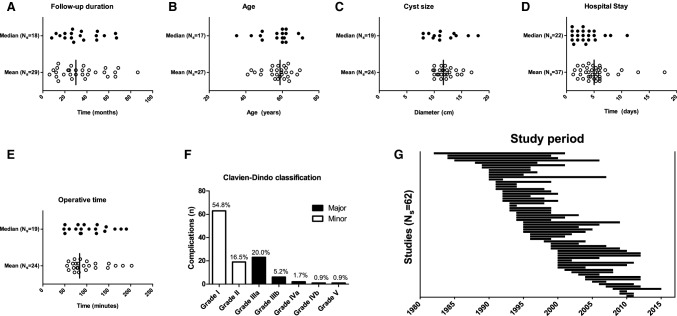
**A** –**E** Analysis of continuous data: reported medians and means, *N*_s_, number of studies. For reported means, the vertical line represents the median of means. **C** Preoperative cyst size, diameter in centimetres. **F** Scoring of post-operative complications according to Clavien–Dindo. **G** Timeframes wherein patients were included (study periods) are shown per study, sorted chronologically on first inclusion

The 62 included studies comprising a total of 1314 patients (Table 1). Studies from 5 different continents were included and most included studies were performed in Europe (Fig. 1B). The median number of patients per study was 17 (total range 3–66). Of all included studies, 5 were prospective cohort studies, 10 were retrospective analyses of prospectively collected data, 28 were retrospective cohort studies and 19 studies did not give an explicit statement on data collection. Publication dates ranged from 1994 to 2017. Study periods ranged between 1982 and 2015 (Fig. 2G). Median follow-up duration was 30 months (IQR 19–48) (Fig. 2A).

**Table 1:** Summary of included studies

#	First author	Year	*N*_p_
1	Ammori	2002	3
2	Andriani	2000	17
3	Ardito	2013	47
4	Bai	2007	44
5	Caetano	2006	12
6	Cappellani	2002	9
7	De Reuver	2017	35
8	Debs	2016	27
9	Descottes	2000	15
10	Diez	1998	10
11	Emmermann	1997	18
12	Fabiani	2005	26
13	Faulds	2010	5
14	Fiamingo	2003	15
15	Gall	2009	61
16	Gamblin	2008	46
17	Gigot	2001	19
18	Gocho	2013	6
19	Hansen	1997	19
20	Hansman	2001	6
21	Heintz	1995	3
22	Hsu	2005	5
23	Kabbej	1996	13
24	Kamphues	2011	43
25	Katkhouda	2000	25
26	Kisiel	2017	48
27	Koea	2008	24
28	Konstadoulakis	2005	9
29	Koperna	1997	10
30	Kornprat	2004	21
31	Kwon	2003	14
32	Lee	2014	29
33	Lolle Noerregaard	2014	29
34	Manterola	2016	41
35	Marks	1998	17
36	Martin	1998	20
37	Martinez-Perez	2016	12
38	Maruyama	2013	16
39	Mazoch	2011	15
40	Mazza	2009	66
41	Morino	1994	11
42	Neri	2006	15
43	Palanivelu	2006	27
44	Pante	2014	7
45	Petri	2002	34
46	Regev	2001	18
47	Robinson	2005	11
48	Roesch Dietlen	1999	7
49	Sasi Szabo	2006	25
50	Schachter	2001	14
51	Scheuerlein	2013	47
52	Sendt	2009	27
53	Tagaya	2003	5
54	Tan	2005	10
55	Tocchi	2002	8
56	Torices	2004	21
57	Torres	2009	13
58	Treckmann	2010	42
59	Van Keimpema	2008	12
60	Wahba	2011	23
61	Wu	2014	30
62	Zacherl	2000	7
	Total	1994–2017	1314

Last name of first author, year of publication

*N*_*p*_ number of included patients per study

Of all included patients, 74% was female and 33% had PLD. Median age at time of operation was 58.7 years (IQR 54.5–62.0) (Fig. 2B). Average preoperative cyst diameter was 11.9 cm (95% CI 11.1–12.7) (Fig. 2C). In 10 studies that did not exclusively operate on solitary cysts, median number of treated cysts was 1.4 (IQR 1.3–2.0; total range 1.2–37.7). Individual study results are presented in Supplementary File 4A–B.

### Efficacy

There were 27 studies that reported the proportion of patients with full or partial symptomatic relief after surgery. Symptomatic relief was based on clinical follow-up data in 25 studies, on a structured telephone interview in one study [14] and on a specific questionnaire in another study [15]. Pooled symptomatic relief was 90.2% (95% CI 84.3–94.9). Symptomatic recurrence during follow-up was 9.6% (95% CI 6.9–12.8). The rate of reintervention for the same cyst was 7.1% (95% CI 5.0–9.4) (Table 2). Mean time until symptomatic recurrence was 16.1 months in 10 patients. Mean time until reintervention was 22.1 months in 13 patients.

**Table 2:** Overall versus PLD outcomes

Outcome	*N*s	Overall				PLD				
		*N*_p_	PE (%)	95% CI	I2 (%)	*N*_s_	*N*_p_	PE (%)	95% CI	I2 (%)
Recurrence	62	1314	9.6	6.9–12.8	68	15	146	33.7*	18.7–50.4	76
Reintervention	56	1176	7.1	5.0–9.4	50	10	109	26.4*	12.6–43.0	69
Complications	60	1276	10.8	8.1–13.9	62	13	129	29.3*	16.0–44.5	69
Major	56	1106	3.3	2.2–4.7	27	13	129	7.2	2.1–14.6	46
Conversions	44	889	4.5	3.2–6.0	0	9	83	8.2	3.2–15.0	0
Mortality	60	1271	1.0	0.5–1.6	0	13	135	2.3	0.4–5.6	0

Asterisk (*): statistically significant difference

*PLD* polycystic liver disease, *N*_*s*_ number of studies, *N*_*p*_ number of patients, *PE* pooled estimate, *CI* confidence interval

### Safety

Conversion from laparoscopic to open surgery during the procedure was necessary in 4.5% (95% CI 3.2–6.0), typically because of intra-operative bleeding, difficult positioning or extensive adhesions. Median hospital stay was 5.0 days (IQR 3.7–6.0) (Fig. 2D). Post-operative complication rate was 10.8% (95% CI 8.1–13.9), generally consisting of either bile leakage, ascites, pleural effusion or infections. Out of 136 reported post-operative complications, 115 could be scored according to the Clavien–Dindo classification (Fig. 2F). Of scored complications, 71.3% were minor and 28.7% were major. Overall, the pooled estimate of having a major complication after surgery was 3.3% (95% CI 2.1–4.7). The pooled estimate of procedure-related mortality was 1.0% (95% CI 0.5–1.6) (Table 2). This was based on a single patient from a series of 9 patients [16]. The patient presented with severe PLD symptoms. After an uneventful in-hospital stay, acute renal insufficiency ensued 20 days after discharge, followed by hepatorenal failure. The patient succumbed 15 days later. Other studies showed no procedure-related mortality.

### Operative technique

Median operative time was 83.5 min (IQR 72–120) (Fig. 2E). The use of omentopexy was explicitly mentioned in 31 studies that included a total of 824 patients. The pooled estimate for use of omentopexy was 14.8% (95% CI 5.8–26.6), with a total range from 0 to 100% between studies. The use of concomitant cholecystectomy was mentioned in 37 studies that included a total of 822 patients. In 21.5% (95% CI 15.8–27.8) of patients, concomitant cholecystectomy was performed; cited reasons were gallstones on image studies or cyst location adjacent to the gallbladder.

### Risk of bias

An evaluation of the quality of individual studies is presented in Table 3, which provides details of risk of bias within studies, as reflected by adjusted Newcastle–Ottawa Scale (NOS) scoring. Overall, median score for ‘selection of study groups’ was 3 out of 4; median score for ‘comparability of groups’ was 0 out of 2 and median score for ‘ascertainment of outcome of interest’ was 3 out of 3. Median of the total NOS-score was 6 out of 9.

**Table 3:**
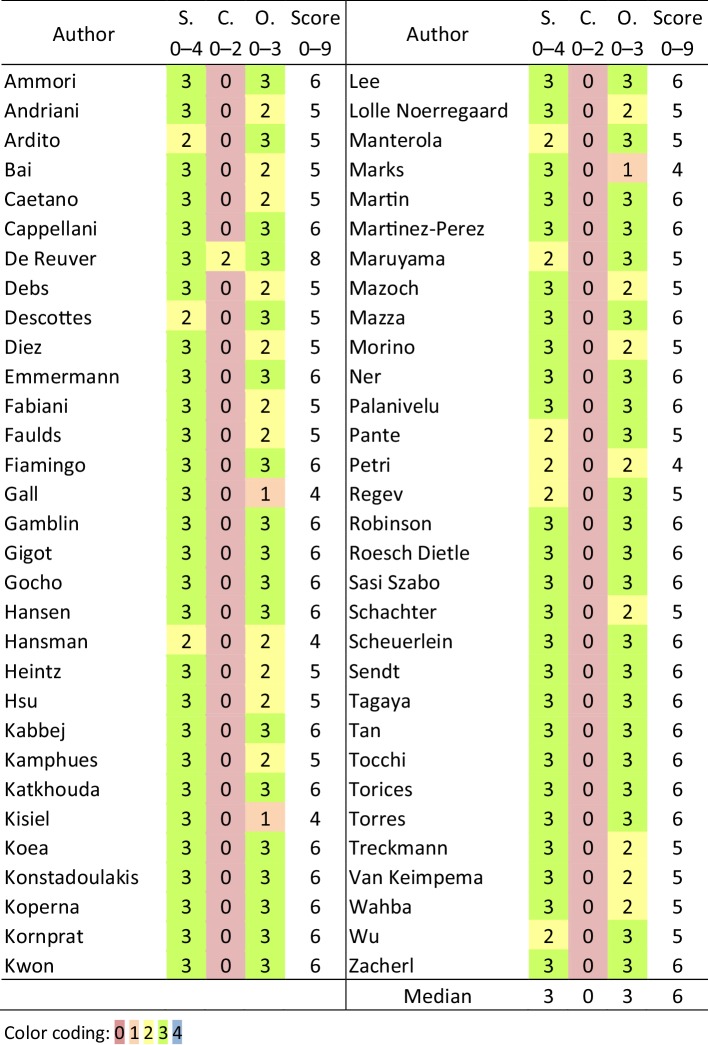
Risk of bias assessment (NOS)

*NOS* Newcastle–Ottawa Scale, *S* selection of the study groups, *C* the comparability of the groups, *O* ascertainment of outcome of interest, *Score* total NOS-Score

### Heterogeneity

Pooled estimates of outcomes were assessed for heterogeneity and publication bias. The I^2^ value for symptomatic relief was 72%, for symptomatic recurrence 68%, for reintervention 50%, for complications 62%, indicating moderate heterogeneity. The *I*^2^ value for intra-operative conversions and for mortality was 0%, indicating negligible heterogeneity.

### Publication bias

**Fig. 3:**
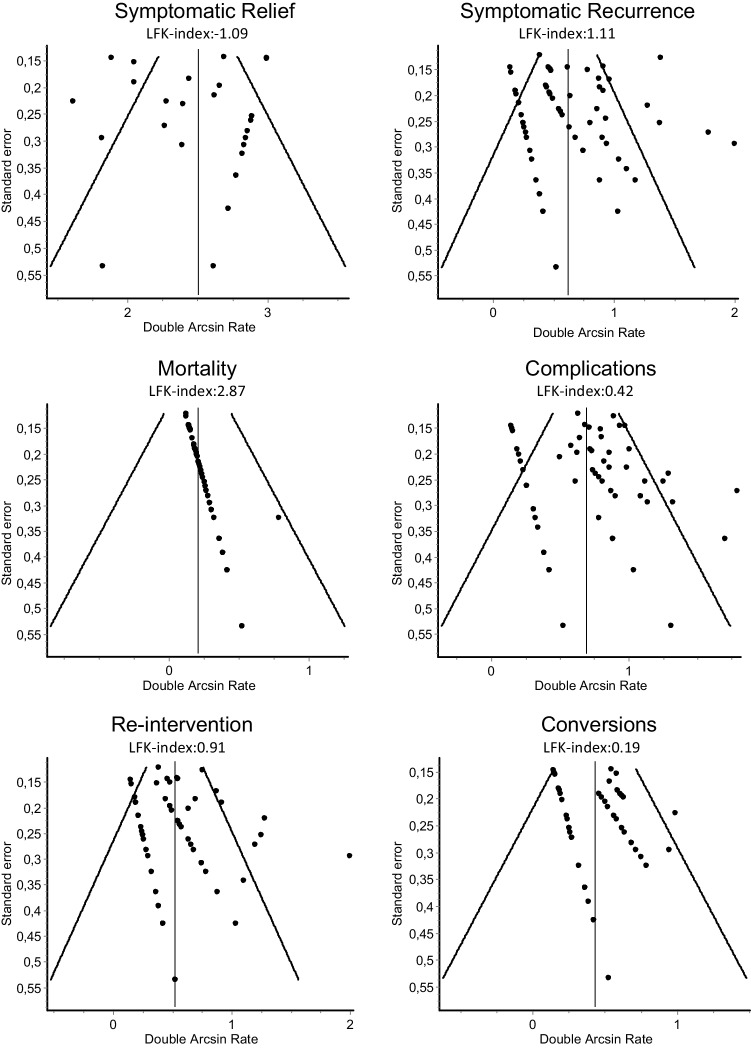
Funnel plots of meta-analysis outcomes. The modelled standard error is plotted against the double arcsine transformed estimates of individual studies. Luis Furuya-Kanamori asymmetry index (LFK-index) is also shown

LFK-index for reintervention was 0.91, for complications 0.42 and for intra-operative conversions 0.19, indicating no asymmetry. LFK-index for symptomatic relief was − 1.09, for symptomatic recurrence 1.11, indicating minor asymmetry. LFK-index for mortality was 2.87, indicating major asymmetry. Funnel plots are shown in Fig. 3.

### Subgroup analyses

#### Polycystic liver disease

**Fig. 4:**
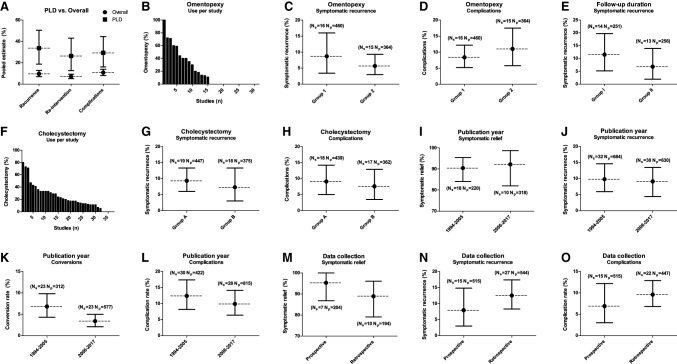
Subgroup analyses. N_s_: number of studies. N_p_: number of patients. Interrupted lines: pooled estimates. Error bars: 95% confidence intervals. **A** Outcomes for the polycystic liver disease (PLD) subgroup and overall results. **B** Percentage of patients that underwent omentopexy per included cohort. **C**, **D** Outcomes for omentopexy subgroups (Group 1: no omentopexy, Group 2: omentopexy). **E** Outcomes for mean follow-up subgroups (Group I: ≤38 months, Group II: >38 months). **F** Percentage of patients that underwent concomitant cholecystectomy per included cohort. **G, H** Outcomes for concomitant cholecystectomy subgroups (Group A: ≤ 21.5%, Group B: > 21.5%). **I**–**L** Outcomes for publication year subgroups (1994–2005 and 2006–2017). **M**–**O** Outcomes for data collection subgroups (prospective and retrospective)

We performed a subgroup analysis of 15 studies that included only PLD patients or reported outcomes of PLD patients separately and compared these to the overall results (Table 2). Symptomatic recurrence and reintervention rates were significantly higher with respective rates of 33.7% (95% CI 18.7–50.4) and 26.4% (95% CI 12.6–43.0). Post-operative complications were more frequent in PLD patients with a pooled estimate of 29.3% (95% CI 16.0–44.5) (Fig. 4A). Out of 37 reported post-operative complications, all could be scored according to Clavien–Dindo. Of scored complications, 70.3% were minor and 29.7% were major. Overall, the pooled estimate of having a major complication after surgery was 7.2% (95% CI 2.1–14.6). Conversion rate and procedure-related mortality did not differ significantly from overall results. Data were insufficient to analyse symptomatic relief in the PLD subgroup.

#### Omentopexy

For analysis of the effect of omentopexy on symptomatic recurrence rates, 31 studies that specified the use of omentopexy were split into two groups. As the median of additional omentopexy was 0%, studies were split accordingly. Group 1: no omentopexy performed. Group 2: omentopexy performed in 1 or more patients (total range 11–100%). For group 1, pooled symptomatic recurrence was 8.7% (95% CI 3.4–16.0). For group 2, it was 5.7% (95% CI 3.0–9.3). In addition, we assessed the effect of omentopexy on post-operative complication rates in the same groups. For group 1, pooled complication rate was 8.4% (95% CI 5.2–12.2). For group 2, it was 11.0% (95% CI 5.8–17.5). In summary, there were no significant differences in pooled estimates of symptomatic recurrence rates and complication rates between groups. Data were insufficient to correct for cyst location and cyst size. (Fig. 4B–D).

#### Concomitant cholecystectomy

For analysis of the effect of concomitant cholecystectomy on symptomatic recurrence rates, 37 studies that specified the use of cholecystectomy were split into two groups. As the median proportion of patients that underwent additional cholecystectomy was 18.2%, studies were divided accordingly. Group A: cholecystectomy in 18.2% of patients or less (total range 0–18.2%). Group B: cholecystectomy in more than 18.2% of patients (total range 21–80%). For group A, pooled symptomatic recurrence was 9.3% (95% CI 6.0–13.3). For group B, it was 7.3% (95% CI 3.0–13.3). Next, we focused on the effect of concomitant cholecystectomy on post-operative complication rates in the same groups. For group A, pooled complication rate was 9.1% (95% CI 5.0–14.2). For group B, it was 7.6% (95% CI 3.5–12.9). These data are consistent with the absence of significant differences in pooled estimates of symptomatic recurrence rates and complication rates between groups (Fig. 4F–H).

#### Follow-up duration

We were interested in the effect of prolonged follow-up on symptomatic recurrence rates. To this end, we selected 27 studies that specified mean follow-up and distinguished into two groups. The median of reported mean follow-up duration was 30 months, and we categorised studies in two groups accordingly. Group I: mean follow-up duration of 30 months or less (total range 6–30 months). Group II: mean follow-up duration of more than 30 months (total range 36–86.4). For group I, pooled symptomatic recurrence was 11.5% (95% CI 5.2–19.7). For group II, it was 6.8% (95% CI 1.9–13.9). Thus, there was no significant effect of length of follow-up after six months on reported symptomatic recurrence rates (Fig. 4E).

#### Publication date

Publication dates ranged between 1994 and 2017, with the year 2005 as the median. Pooled symptomatic relief for studies published from 1994 to 2005 was 90.4% (95% CI 84.0–95.4), and for studies published from 2006 to 2017 it was 92.2% (95% CI 82.0–98.7). Symptomatic recurrence for studies published from 1994 to 2005 was 9.8% (95% CI 5.9–14.6) and for studies published from 2006 to 2017 it was 9.1% (95% CI 4.4–13.5). Next, we assessed the effect of publication date on conversion rates. In studies published from 1994 to 2005, the pooled conversion rate was 6.8% (95% CI 4.3–9.8) and for studies published from 2006 to 2017 it was 3.4% (95% CI 2.1–5.0). It must be noted that there were four studies with a conversion rate of 10% or higher and all were published before 2006 [16–19]. In studies published from 1994 to 2005, the pooled complication rate was 12.4% (95% CI 8.2–17.4) and for studies published from 2006 to 2017 it was 9.9% (95% CI 6.4–14.1). In studies published from 1994 to 2005, the median hospital stay was 5.3 days (IQR 4.0–6.3). In studies published from 2006 to 2017, the median hospital stay was 4.7 days (IQR 3.5–5.7), medians were not significantly different (*p* = 0.23). We can conclude that there were no significant effects of publication date on outcomes (Fig. 4I–L).

#### Data collection

To assess the effect of original study design on our primary outcomes, we performed a subgroup analysis on 15 studies that performed data collection prospectively and 27 studies that did retrospectively. In the prospective subgroup, symptomatic relief was 95.3% (95% CI 86.8–100.0%), symptomatic recurrence was 7.9% (95% CI 3.0–14.8) and complication rate was 6.9% (95% CI 3.0–12.2). In the retrospective subgroup, symptomatic relief was 88.9% (95% CI 79.1–96.1), symptomatic recurrence was 12.5% (95% CI 8.3–17.4) and complication rate was 9.6% (95% CI 6.8–12.9). We can state that there were no significant effects of data collection on outcomes (Fig. 4M–O).

## Discussion

### Efficacy and safety

This systematic review describes the safety and efficacy of laparoscopic fenestration in 1314 patients reported in 62 individual studies. We document that laparoscopic fenestration of large, symptomatic cysts is effective and results in symptomatic relief in the large majority of patients. Symptomatic recurrence after fenestration is low (9.6%) as is the reintervention rate for the same cyst (7.1%). Omentopexy after cyst fenestration did not improve efficacy, but also was not associated with a higher complication rate.

Laparoscopic fenestration appears to be a safe procedure and while procedure-related complications do occur in 11% of patients, scoring according to Clavien–Dindo shows that these are mostly minor and amenable to treatment. We were unable to assess the relation between pre-surgical cyst size, complication rate and recurrence rate. Concomitant cholecystectomy is feasible, but does not contribute to the overall success of the procedure but similarly does not result in a higher complication rate.

The average interval between surgery and symptomatic recurrence was 16 months, and mean time until reintervention was 22 months. This interval should be interpreted very carefully because of the small sample size, but underscores the need for long-term follow-up when investigating cyst recurrence in future studies.

Patients with PLD may possess one or more large cysts against the background of multiple smaller cysts in surrounding liver. Symptoms in PLD may be attributed to these large cysts and it may be tempting to perform laparoscopic fenestration here. We found that this subgroup is at a high risk for complications and that long-term symptomatic relief is less well achieved. Potential causes of the elevated risk of complications are the changes in hepatic anatomy in PLD and the use of extensive fenestration, with some studies fenestrating over 30 cysts per patient [16, 20]. The elevated recurrence rate is probably related to the different natural history of PLD and large solitary cysts. Hepatic cysts, regardless whether they are solitary or multiple, arise as a result from inactivation of 2 alleles from PLD genes. PLD is a genetic disorder and patients have a germline mutation in one of the PLD genes and must acquire only one additional somatic mutation to develop cysts. Patients with solitary large cysts need to acquire somatic mutations on 2 PLD genes to develop the phenotype [3]. Thus, the risk for recurrence is low in these patients. This contrasts with the situation in PLD where the liver volume increases with 1.8% every 6–12 months. As a consequence, the natural growth of PLD will rapidly overtake the potential volume-curtailing effect of laparoscopic fenestration of a single, albeit large, cyst. The implication is that the threshold for laparoscopic fenestration in PLD must be high in view of the limited long-term efficacy and higher risks.

Percutaneous aspiration sclerotherapy is a valid alternative strategy for large simple hepatic cysts. A recent systematic review found that aspiration sclerotherapy reduces symptoms in 72–100% while symptoms disappeared in 56–100% of patients. Aspiration sclerotherapy comes with complications such as pain, ethanol intoxication, cyst bleeding and rarely cyst infections [21]. It is essential to understand the dynamics of fluid reaccumulation and disappearance after aspiration sclerotherapy to appreciate the merits of the procedure. Within days after complete evacuation of the cyst using aspiration sclerotherapy, cyst fluid reaccumulates only to disappear slowly over (at least) 26 weeks [22]. As a corollary, aspiration sclerotherapy takes months to achieve its full effect, compared to the immediate effect of fenestration. Despite these differences, it still needs to be determined which treatment is superior or which patient subgroup has the most benefit from either procedure. As percutaneous aspiration sclerotherapy and laparoscopic fenestration have never been compared directly in a controlled setting, we believe that a randomised trial that focuses primarily on symptomatic relief and symptomatic recurrence should be conducted. Subgroup analyses might elucidate patient-related factors that make either procedure better suited.

### Surgical technique

The question here is whether the evolution of laparoscopic fenestration is complete. In our dataset, we did not find a significant change in rates of efficacy, complications, conversions to laparotomy or length of hospital stay over time. Although conversion rates above 10% only occurred before 2006. The basic surgical technique used is straightforward and entails laparoscopy, aspiration of cyst fluid first and finally wide deroofing of the cyst wall (near the transition zone between cyst wall and normal hepatic parenchyma). There are innovations such as the use of robot-assisted laparoscopic fenestration for giant hepatic cysts [23], single incision laparoscopic surgery [24–30] or 3D-vision supported surgery [31]. In addition, the use of indocyanine green fluorescent imaging intra-operatively may facilitate better assessment of bile duct communication or identification of bile duct injuries [32–36]. However, the additive value of these techniques for cyst fenestration remains unclear.

Cyst recurrence is an issue and is thought to result from incomplete deroofing or development of a false lumen by adjacent tissues [37, 38]. To reduce recurrence risk, omentopexy is advocated in view of the hypothesis that omental tissue resorbs fluid and keeps the residual cavity open. Some authors cite specific indications to perform omentopexy such as a small exposed cyst wall, intrahepatic cysts, cyst size > 10 cm, cysts located posteriorly or in segment 7 and 8 or if < 50% of cyst wall can be resected [15, 39–45]. Other researchers refrain from omentopexy because of questionable evidence, similar recurrence rates without omentopexy, additional complications (e.g. omental bleeding) or extension of operating time [16, 46–49].

Our systematic review did not identify advantages or disadvantages of omentopexy as adjunct to the surgical procedure. One caveat is that the data were limited and no correction for cyst size and cyst location could be made. We included only studies that explicitly mentioned omentopexy in the subgroup analysis and it is possible that we missed data from studies that used the procedure but did not report that. Randomised clinical trial data are lacking but a single retrospective study compared fenestration with or without omentopexy and did not report a significant benefit [49]. In view of the limited benefit, the customary use of omentopexy with laparoscopic fenestration is questionable. Other options are in development to curtail cyst recurrence after deroofing such as ethanol sclerotherapy [50–53], argon beam coagulation [54, 55] or wide electrocoagulation [56], but evidence to support their use is limited and the provided data were not sufficient to perform a subgroup analysis of these techniques.

### Strengths and limitations of the study

There are a number of strengths and limitations that result from the very nature of a systematic review. The compliance with the recommendations of the PRISMA and MOOSE guidelines is a major strength of our systematic review. This included a pre-published protocol, an up-to-date extensive literature search, independent screening of all references by two authors and independent risk of bias assessment of included studies by two authors. Data extraction was checked for errors by random sampling of 6 studies by a second investigator and was found 100% accurate. Contact with the corresponding authors of the included studies for additional information provided an extra inclusion. We excluded studies with a mean or median follow-up < 6 months to reduce biases in reported recurrence rates. Selection bias was reduced by excluding case series and all articles were methodically checked for presence of duplicate datasets. A limitation of our review is that we could not include some studies because of language restrictions and unavailable full-text articles. This resulted in exclusion of some substantial Russian [57, 58], Ukrainian [59], Romanian [60], Hungarian [61] and Chinese [62, 63] cohorts, which is a possible source of bias and may result in lower generalisability in other countries. In addition, an important question is if the location of the treated hepatic cyst correlates with a different clinical response. It has been reported that unfavourably located cysts have a higher tendency for recurrence [17]. Unfortunately, the provided data were not sufficient to analyse this question in a subgroup analysis.

In our risk of bias assessment, studies scored well on selection of the study groups and ascertainment of outcome of interest. However, studies scored very low on comparability of groups, as most studies did not include a control group. The implication is that the data collection resulted in a robust dataset but that comparison to untreated patients and correction for centre-dependent biases is not possible.

We observed moderate heterogeneity for the outcomes symptomatic relief, symptomatic recurrence, reintervention and complication rate. This is in part attributable to the diverse patient populations (PLD, solitary cysts or both). Our subgroup analyses established that omentopexy, cholecystectomy, follow-up duration, publication date and data collection did not significantly affect the results and are an improbable cause of heterogeneity. Remaining causes of heterogeneity, that could not be assessed, are clinical diversity (e.g. centre, surgical expertise) and methodological diversity (e.g. study design, reporting).

Most outcomes had an LFK-index demonstrating minor or no asymmetry in the publication bias assessment, except for mortality. In theory, this could indicate that studies with high mortality were less likely to be published. However, as most studies had a prevalence of 0% and the one reported procedure-related death occurred in a small cohort, the pooled mortality rate and LFK-index are probably overestimated.

No randomised controlled trials were included, and most included studies used patient records or prospective databases. Only few studies used a validated questionnaire to assess symptoms and none used specific questionnaires such as the PLD-Q or POLCA [3]. In addition, not all studies had a clear definition of symptomatic recurrence and it is unclear if imaging had been performed for all patients during follow-up. In order to address this issue, we pooled patients with recurrent symptoms with evidence of radiological recurrence and patients with recurrent symptoms. By pooling both categories it is possible that we included patients with recurrent symptoms without radiological recurrence. This could have affected our results. However, in included studies, only 3 out of 1203 patients had recurrent symptoms without radiological recurrence. We suggest that any future studies use validated questionnaires and standard imaging techniques at pre-set time points.

## Conclusions

In conclusion, this systematic review provides evidence that laparoscopic fenestration is an effective treatment for symptomatic simple hepatic cysts with a low symptomatic recurrence rate. The symptomatic recurrence rate and risk of complications are significantly higher in PLD patients.

## Electronic supplementary material

Below is the link to the electronic supplementary material.

Supplementary material 1 (DOCX 114 KB)
