# Supplementary material for: Clinical response after laparoscopic fenestration of symptomatic hepatic cysts: a systematic review and meta-analysis
Source: Surg Endosc. 2018 Oct 17;33(3):691–704. doi: 10.1007/s00464-018-6490-8 (PMC6394680; doi:10.1007/s00464-018-6490-8)
Supplement: Supplementary file 1 — Supplementary material 1 (DOCX 114 KB) [file 464_2018_6490_MOESM1_ESM.docx]

**Supplementary file 1: MOOSE checklist**

From: Stroup DF, Berlin JA, Morton SC, et al. Meta-analysis of observational studies in epidemiology: a proposal for reporting. Meta-analysis Of Observational Studies in Epidemiology (MOOSE) group. *JAMA* 2000; 283(15):2008-12.

| **Section/Topic** | **Brief description of how the criteria were handled in the meta-analysis** |
| --- | --- |
| **Background** | |
| 1. Problem definition | Laparoscopic fenestration is one of the treatment options for symptomatic simple hepatic cysts, either solitary or in context of polycystic liver disease (PLD), but indications, effect size and surgical techniques are under debate. |
| 1. Hypothesis statement | Laparoscopic fenestration is effective and safe in selected populations. |
| 1. Description of study outcomes | Symptomatic relief or symptomatic recurrence |
| 1. Type of exposure or intervention used | Laparoscopic fenestration combines cyst fluid aspiration, followed by excision of extra-hepatic cyst wall in a single laparoscopic procedure |
| 1. Type of study designs used | Cohort studies and clinical trials |
| 1. Study population | Adults with large, symptomatic, simple hepatic cysts |
| **Search strategy** | |
| 1. Qualifications of searchers (eg, librarians and investigators) | The credentials of the three investigators are indicated in the authors list and the librarian who performed the study is mentioned in the acknowledgements section. |
| 1. Search strategy, including time period included in the synthesis and keywords | See methods for search strategy. Period: inception to 18 July 2017, without any restrictions. |
| 1. Effort to include all available studies, including contact with authors | Authors were emailed. |
| 1. Databases and registries searched | MEDLINE, Embase, Web of Science and the Cochrane Library |
| 1. Search software used, name and version, including special features used (eg, explosion) | PubMed. OvidSP. |
| 1. Use of hand searching (eg, reference lists of obtained articles) | Bibliographies of included articles were hand searched for additional references. |
| 1. List of citations located and those excluded, including justification) | Details of the literature search process are outlined in the PRISMA flow chart. The citation list of excluded articles is available upon request. |
| 1. Method of addressing articles published in languages other than English | Articles in the following languages were included: Dutch, English, French, German, Italian and Spanish. |
| 1. Method of handling abstracts and unpublished studies | Abstracts and unpublished studies were excluded. |
| 1. Description of any contact with authors | When full-text could not be obtained, we contacted authors via email. When additional data was necessary, we contacted authors via email. |
| **Methods** | |
| 1. Description of relevance or appropriateness of studies assembled for assessing the hypothesis to be tested | Detailed inclusion and exclusion criteria are described in the methods section of the article. |
| 1. Rationale for the selection and coding of data (eg sound clinical principles or convenience) | Data extraction sheets were developed (available on request). Extracted data was related to bibliographic details of included studies, patient characteristics, relevant outcomes, study design and quality assessment |
| 1. Documentation of how data were classified and coded (multiple raters, blinding and inter-rater reliability) | Detailed documentation can be found in the methods section of the article. |
| 1. Assessment of confounding (eg, comparability of cases and controls in studies where appropriate) | Confounders were searched for with pre-specified subgroup analyses |
| 1. Assessment of study quality, including blinding of quality assessors; stratification or regression on possible predictors of study results. | We used the Newcastle-Ottawa scale to assess quality |
| 1. Assessment of heterogeneity | We used the I^2^ value to assess heterogeneity |
| 1. Description of statistical methods (eg, complete description of fixed or random effects models, justification of whether the chosen models account for predictors of study results) in sufficient detail to be replicated | Pooled estimates and 95% confidence intervals were calculated with a random effects model). Detailed documentation can be found in the methods section of the article. |
| 1. Provision of appropriate tables and graphics | PRISMA flow chart is presented in article. Table 1 summarizes included articles. |
| **Results** | |
| 1. Graphic summarizing individual study estimates and overall estimate | Individual study results are added as supplementary files. Overall estimates are presented in the Results section. |
| 1. Table giving descriptive information for each study included | Table 1 and supplementary files. |
| 1. Results of sensitivity testing (eg, subgroup analysis) | Subgroup analyses can be found in Figure 4, detailed descriptions are presented in Results section of the article. |
| 1. Indication of statistical uncertainty of findings | For pooled estimates, 95% confidence intervals are presented together with I^2^ values. For medians, IQR and/or total range is presented. |
| **Discussion** | |
| 1. Quantitative assessment of bias (eg, publication bias) | Results of subgroup analyses are discussed in the results and discussion section. Risk of publication bias is described in results section and funnel plots are shown in Figure 3. |
| 1. Justification for exclusion (eg, exclusion of non-English-language citations) | Detailed documentation can be found in the Methods section of the article. |
| 1. Assessment of quality of included studies | NOS scores for individual studies are shown in Table 3. Implications are described in the discussion section of the article. |
| **Conclusions** | |
| 1. Consideration of alternative explanations for observed results | Confounders and biases are described in the discussion section of the article. |
| 1. Generalization of the conclusions (ie, appropriate data presented and within the domain of the literature review) | Generalizability is described in the discussion section of the article. |
| 1. Guidelines for future research | We suggest that any future studies use validated questionnaires and standard imaging techniques at pre-set time points. We propose a randomized clinical trial comparing percutaneous aspiration sclerotherapy and laparoscopic fenestration |
| 1. Disclosure of funding source | No financial disclosures |

**Supplementary file 2: Search terms**

| **Pubmed MEDLINE** | | |
| --- | --- | --- |
| **PICO format** | **No.** | **Search terms** |
| 1. Patient | #1 | (“Liver diseases”[MeSH] AND “Cysts”[MeSH Terms]) OR “Polycystic liver disease”[Supplementary Concept] |
|  | #2 | ((hepatic[tiab] OR liver[tiab] OR livers[tiab]) AND (cyst[tiab] OR cysts[tiab] OR cystic[tiab])) OR polycystic liver[tiab] OR liver polycystic[tiab] OR PCLD[tiab] |
|  | #3 | #1 OR #2 |
| 2. Intervention | #4 | "Laparoscopy"[Mesh] OR "Video-Assisted Surgery"[Mesh:NoExp] OR "Minimally Invasive Surgical Procedures"[Mesh:NoExp] |
|  | #5 | Fenestration*[tiab] OR deroofing*[tiab] OR de-roofing*[tiab] OR marsupialisation*[tiab] OR marsupialization*[tiab] OR minimally invasive surg*[tiab] OR minimally invasive procedure*[tiab] OR minimally invasive technique*[tiab] OR minimally invasive method*[tiab] OR laparoscop*[tiab] OR video assisted surger*[tiab] OR video assisted procedure*[tiab] OR videolaparoscop*[tiab] OR laparoendoscopic[tiab] |
|  | #6 | #4 OR #5 |
| 3. Comparator |  | *Not specified* |
| 4. Outcome |  | *Not specified* |
| 5. PICO | #7 | #3 AND #6 |
| **OvidSP Embase** | | |
| **PICO format** | **No.** | **Search terms** |
| 1. Patient | #1 | liver cyst/ OR liver polycystic disease/ |
|  | #2 | (((hepatic OR liver OR livers) AND (Cyst OR cysts OR cystic))OR Polycystic liver OR liver polycystic OR PCLD).ti,ab,kw. |
|  | #3 | #1 OR #2 |
| 2. Intervention | #4 | laparoscopy/ OR hand assisted laparoscopy/ OR laparoendoscopic single site surgery/ OR laparoscopic surgery/ OR exp minimally invasive procedure/ |
|  | #5 | (Fenestration* OR deroofing* OR de-roofing* OR marsupialisation* OR marsupialization* OR minimally invasive surg* OR minimally invasive procedure* OR minimally invasive technique* OR minimally invasive method* OR laparoscop* OR video assisted surger* OR video assisted procedure* OR videolaparoscop* OR laparoendoscopic).ti,ab,kw. |
|  | #6 | #4 OR #5 |
| 3. Comparator |  | *Not specified* |
| 4. Outcome |  | *Not specified* |
| 5. PICO | #7 | #3 AND #6 |
| **Cochrane Library & Web of Science** | | |
| **PICO format** | **No.** | **Search terms** |
| 1. Patient | #1 | ((hepatic OR liver OR livers) AND (cyst OR cysts OR cystic)) OR “polycystic liver” OR “liver polycystic” OR PCLD |
| 2. Intervention | #2 | fenestration* OR deroofing* OR “de-roofing*” OR marsupialisation* OR marsupialization* OR “minimally invasive surg*” OR “minimally invasive procedure*” OR “minimally invasive technique*” OR “minimally invasive method*” OR laparoscop* OR “video assisted surger*” OR “video assisted procedure*” OR videolaparoscop* OR laparoendoscopic |
| 3. Comparator |  | *Not specified* |
| 4. Outcome |  | *Not specified* |
| 5. PICO | #3 | #1 AND #2 |

**Supplementary File 3: Adjusted Newcastle-Ottawa scale for cohort studies**

| **Selection (0-4)** |
| --- |
| **1) Representativeness of the exposed cohort** |
| a) truly representative of the average community (1 point) |
| b) somewhat representative of the average community (1 point) |
| c) selected group of users e.g. nurses, volunteers |
| d) no description of the derivation of the cohort |
| **2) Selection of the non exposed cohort** |
| a) drawn from the same community as the exposed cohort (1 point) |
| b) drawn from a different source |
| c) no description of the derivation of the non exposed cohort |
| d) not applicable |
| **3) Ascertainment of exposure** |
| a) secure record (e.g. surgical records) (1 point) |
| b) structured interview (1 point) |
| c) written self report |
| d) no description |
| **4) Demonstration that outcome of interest was not present at start of study** |
| a) yes (symptomatic patients) (1 point) |
| b) no (asymptomatic patients) |
| c) no description |
| **Comparability (0-2)** |
| **1) Comparability of cohorts on the basis of the design or analysis** |
| a) study controls for median cyst size (1 point, max. 2) |
| b) study controls for cyst location (1 point, max. 2) |
| c) symptomatic patients (1 point, max. 2) |
| c) not applicable |
| **Outcome (0-3)** |
| **1) Assessment of outcome** |
| a) independent blind assessment OR conformation by secure records (1 point) |
| b) record linkage (1 point) |
| c) self report |
| d) no description |
| **2) Was follow-up long enough for outcomes to occur** |
| a) >12 months (1 point) |
| b) > 6 months |
| **3) Adequacy of follow-up of cohorts** |
| a) complete follow-up - all subjects accounted for (1 point) |
| b) >80% follow-up, or description provided of those lost |
| c) follow-up rate < 80% and no description of those lost |
| d) no statement |

**Supplementary File 4A: Individual study results (characteristics)**

| **Study (subgroups)** | **Year** | **Design** | **Study period** | **Location** | **N_p_** | **PLD**  **(n)** | **Female**  **(n)** | **Age (years)** | **Cyst diameter**  **(cm)** | **Operated cysts**  **(N_p_)** | **Follow-up**  **(months)** |
| --- | --- | --- | --- | --- | --- | --- | --- | --- | --- | --- | --- |
| Ammori^1^ | 2002 | RCS | 1992-1999 | UK | 3 | 1 | NS | NS | NS | NS | NS |
| Andriani^2^ | 2000 | RCS | 1993-1998 | Argentina | 17 | 0 | 11 | NS | Mean 9.8 cm (5-30cm) | Total 24 | Mean 23 |
| Ardito^3^ | 2013 | RAPCD | 2000-2010 | Italy | 47 | 19 | 10 | Mean 57.6 | Mean 13 cm (5-22) | Total 96 | Median 67 |
| Bai^4^ | 2007 | RCS | 1998-2004 | China | 44 | 10 | 30 | Mean 57 | Mean 12.3 (6-23) | NS | Mean 57 |
| Caetano^5^ | 2006 | NS | 1994-1999 | Brazil | 12 | 3 | 12 | Mean 56 | Median 9.8 (6-15) | NS | NS |
| Cappellani^6^ | 2002 | NS | 1992-2000 | Italy | 9 | 1 | 9 | NS | Mean 11.5 (8-16) | NS | Mean 42 |
| Debs^7^ | 2016 | RCS | 2000-2012 | France | 27 | NS | NS | NS | NS | NS | Mean 24 |
| Descottes^8^ | 2000 | RCS | 1991-1999 | France | 15 | 4 | 14 | Mean 54 | 15.7 (8.3-23) | NS | Mean 14 |
| De Reuver^9^ | 2017 | RAPCD | 2000-2013 | Australia | *35* | 2 | 30 | Mean 63.7 | Mean 14 cm. Median 12 cm. | NS | Median 49,64.  Mean 65,79 |
| Diez^10^ | 1998 | NS | 1992-1996 | Argentina | 10 | 1 | NS | Mean 43 | NS | NS | NS |
| Emmermann^11^ | 1997 | NS | 1991-1994 | Germany | 18 | 0 | 18 | Mean 57 | Mean 12 (9-18) | 18 | Mean 19 |
| Fabiani^12^ | 2005 | RCS | 1989-2001 | France | 26 | 0 | 35 | Median 68.8 | Mean 11 (5.5-20) | NS | Mean 68,7 |
| Faulds^13^ | 2010 | PCS | 2009-2010 | Canada | 5 | NS | 6 | Mean 63.2 | Mean 11.3 (7-16) | NS | Mean 9,6 |
| Fiamingo^14^ | 2003 | RCS | 1996-2002 | Italy | 15 | 6 | 13 | Median 57 | Median 9 (7-13) in No PLD  Median 11 (10-13) in PLD | Total 18 | Median 34 |
| Gall (PLD)^15^ | 2009 | RAPCD | 1985-2006 | UK | 13 | 13 | NS | Median 49 | Median 10 (4-18) | NS | NS |
| Gall (No PLD)^15^ | 2009 | RAPCD | 1985-2006 | UK | 48 | 0 | NS | Median 60 | Median 11 (2-30) | NS | NS |
| Gamblin^16^ | 2008 | RAPCD | 2001-2008 | USA | 46 | 45 | 40 | Median 60 | Median 13 (2-21) | NS | Mean 13 |
| Gigot^17^ | 2001 | NS | 1984-1999 | Belgium | 19 | 2 | 18 | Median 57 | Mean 13 (8-30) | NS | Mean 38,5 |
| Gocho^18^ | 2013 | NS | 2010-2011 | Japan | 6 | NS | 6 | Median 60 | Median 11.1 (10.0-15.2) | NS | Median 15,5 |
| Hansen^19^ | 1997 | PCS | 1990-1996 | USA | 19 | 2 | 16 | Mean 65 | Mean 15 (7-25) in No PLD  Mean 9 (6-16) in multiple cysts | NS | Mean 30 |
| Hansman^20^ | 2001 | RCS | 1984-2000 | USA | 6 | 0 | NS | NS | NS | NS | NS |
| Heintz^21^ | 1995 | NS | 1993-1994 | Germany | 3 | NS | 3 | Mean 59.7 | Mean 12.3 (8-16) | NS | Median 8  Mean 6 |
| Hsu^22^ | 2005 | NS | 1996-2001 | Taiwan | 5 | NS | 6 | Mean 59.5 | Mean 16.8 (10-20) |  | Mean 48 |
| **Study (subgroups)** | **Year** | **Design** | **Study period** | **Location** | **N_p_** | **PLD**  **(n)** | **Female**  **(n)** | **Age (years)** | **Cyst diameter**  **(cm)** | **Operated cysts**  **(N_p_)** | **Follow-up**  **(months)** |
| Kabbej^23^ | 1996 | NS | 1991-1994 | France | 13 | 13 | NS | Median 49 | NS | Median 32  (range 18-58) | Median 26 |
| Kamphues^24^ | 2011 | RAPCD | 2002-2008 | Germany | 43 | 7 | 41 | Median 48 | Median 8 (2-27) | Median 1  (range 1-7) | Median 49 |
| Katkhouda (PLD)^25^ | 2000 | NS | 1990-1997 | USA | 9 | 9 | 6 | Median 43 | Median 8 (4-16) | NS | NS |
| Katkhouda (No PLD)^25^ | 2000 | NS | 1990-1997 | USA | 16 | 0 | 12 | Median 35 | Median 14 (7-22) | 16 | NS |
| Kisiel^26^ | 2017 | RAPCD | 2000-2012 | UK | 48 | NS | 39 | Median 71 | NS | NS | Median 66,2 |
| Koea (PLD)^27^ | 2008 | RAPCD | 2000-2006 | New Zealand | 12 | 12 | 12 | Median 62 | NS | NS | Median 26 |
| Koea (No PLD)^27^ | 2008 | RAPCD | 2000-2006 | New Zealand | 12 | 0 | 11 | Median 62 | Median 18 (11-52) | 12 | Median 20 |
| Konstadoulakis^28^ | 2005 | RCS | 2000-2002 | Greece | 9 | 9 | 9 | Mean 69.6 | NS | Mean 37,7  (range 18-64) | Mean 25,8 |
| Koperna^29^ | 1997 | RCS | 1990-1995 | Austria | 10 | 5 | NS | NS | NS | NS | NS |
| Kornprat^30^ | 2004 | NS | 1994-2003 | Austria | 21 | 8 | NS | NS | NS | NS | Median 27 |
| Kwon^31^ | 2003 | NS | 1994-2001 | Japan | 14 | NS | 10 | Mean 62 | NS | Total 19 | Median 56 |
| Lee^32^ | 2014 | RCS | 2004-2012 | South Korea | 29 | NS | NS | NS | NS | NS | NS |
| Lolle Noerregaard^33^ | 2014 | RCS | 2007-2012 | Denmark | 29 | NS | NS | Median 61 | NS | NS | Median 28 |
| Manterola^34^ | 2016 | PCS | 2008-2015 | Chile | 41 | NS | 31 | NS | Mean 10 (6-21) | Total 52 | Median 35 |
| Marks^35^ | 1998 | NS | 1989-1996 | France | 17 | NS | NS | NS | NS | NS | NS |
| Martin (PLD)^36^ | 1998 | RCS | 1988-1997 | UK | 7 | 7 | NS | NS | NS | NS | Mean 37 |
| Martin (No PLD)^36^ | 1998 | RCS | 1988-1997 | UK | 13 | 0 | NS | NS | NS | NS | Mean 25 |
| Martinez-Perez^37^ | 2016 | RCS | 2004-2012 | Spain | 12 | 12 | NS | NS | NS | NS | NS |
| Maruyama^38^ | 2013 | RCS | 2000-2011 | Japan | 16 | 0 | NS | NS | NS | 16 | NS |
| Mazoch^39^ | 2011 | RCS | 1995-2009 | USA | 15 | 0 | NS | NS | Mean 15.32 (6-26) | 15 | NS |
| Mazza (PLD)^40^ | 2009 | RAPCD | 1990-2007 | Argentina | 20 | 20 | NS | NS | NS | NS | NS |
| Mazza (No PLD)^40^ | 2009 | RAPCD | 1990-2007 | Argentina | 46 | 0 | NS | NS | NS | NS | NS |
| Morino (PLD)^41^ | 1994 | NS | 1990-1992 | Italy | 7 | 7 | NS | Mean 54.57 | NS | NS | NS |
| Morino (No PLD)^41^ | 1994 | NS | 1990-1992 | Italy | 4 | 0 | 2 | Mean 57.75 | Mean 14.25 (9-14) | 4 | NS |
| Neri (PLD)^42^ | 2006 | NS | 1999-2003 | Italy | 3 | 3 | NS | NS | NS | NS | NS |
| Neri (No PLD)^42^ | 2006 | NS | 1999-2003 | Italy | 12 | 0 | NS | NS | NS | NS | NS |
| Palanivelu^43^ | 2006 | PCS | 1995-2006 | India | 27 | 0 | 5 | Mean 48.6 | Median 16.2 (5.4-42.6) | Total 27 | Mean 86,4 |
| Pante^44^ | 2014 | NS | 2006-2011 | Italy | 7 | 7 | 5 | Mean 59.4 | NS | NS | NS |
| **Study (subgroups)** | **Year** | **Design** | **Study period** | **Location** | **N_p_** | **PLD**  **(n)** | **Female**  **(n)** | **Age (years)** | **Cyst diameter**  **(cm)** | **Operated cysts**  **(N_p_)** | **Follow-up**  **(months)** |
| Petri^45^ | 2002 | RCS | 1982-2001 | Hungary | 34 | NS | NS | NS | Mean 10.03 ± 3 | NS | Mean 12 |
| Regev^46^ | 2001 | RCS | 1993-1999 | USA | 18 | NS | NS | NS | NS | NS | NS |
| Robinson^47^ | 2005 | RCS | 1995-2003 | USA | 11 | 11 | 10 | Mean 41 ± 7 | (1-10) | NS | Mean 41 |
| Roesch Dietlen^48^ | 1999 | RCS | 1992-1998 | Mexico | 7 | 0 | 5 | Mean 61 | (12-18) | 7 | Mean 26,4 |
| Sasi Szabo^49^ | 2006 | NS | 1995-2005 | Hungary | 25 | 4 | 16 | Mean 54.4 | Mean 6.9 (3.5-20) | NS | Median 48,7 |
| Schachter^50^ | 2001 | RAPCD | 1996-1999 | Israel | 14 | 2 | NS | NS | NS | NS | Mean 30 |
| Scheuerlein^51^ | 2013 | RCS | 2000-2010 | Germany | 47 | NS | NS | NS | NS | Mean 2  (range 1-9) | NS |
| Sendt^52^ | 2009 | RAPCD | 1995-2004 | Germany | 27 | 4 | 24 | Median 60 | Mean 10 (6-20) | Mean 1,3  (range 1-5) | Mean 56 |
| Tagaya^53^ | 2003 | NS | 1993-1999 | Japan | 5 | 0 | 1 | Mean 63 | Mean 10 (7-18) | 5 | Mean 66 |
| Tan^54^ | 2005 | RCS | 1992-2000 | Singapore | 10 | 0 | NS | NS | Mean 12.8 (6-18) | 10 | Median 20 |
| Tocchi^55^ | 2002 | RCS | 1994-1999 | Italy | 8 | 0 | NS | NS | NS | 8 | NS |
| Torices^56^ | 2004 | RCS | 2000-2004 | Mexico | 21 | NS | 15 | Mean 62 | Mean 12 (8-15) | NS | Mean 36 |
| Torres^57^ | 2009 | NS | 2003-2006 | Brazil | 13 | 2 | 11 | Mean 48.3 | Mean 11.3 (9.5-17) in No PLD  Mean 10.6 (9.2-12.1) in PLD | NS | Mean 36 |
| Treckmann^58^ | 2010 | RCS | 1999-2007 | Germany | 42 | 4 | NS | NS | Mean 10.8 (6-18) | NS | NS |
| Van Keimpema^59^ | 2008 | PCS | 2005-2007 | Netherlands | 12 | 12 | 12 | Mean 44.9 | NS | NS | Median 12 |
| Wahba^60^ | 2011 | RCS | 1999-2009 | Germany | 23 | NS | 19 | Mean 68.09 | Mean 12.2 ± 3.2 | NS | Mean 59 |
| Wu (SILS)^61^ | 2014 | RCS | 2009-2011 | China | 15 | NS | 8 | Mean 60.93 | Mean 11.67 ± 3.66 | NS | Mean 12,26 |
| Wu (standard)^61^ | 2014 | RCS | 2009-2011 | China | 15 | NS | 9 | Mean 58.67 | Mean 11.27 ± 2.22 | NS | Mean 14 |
| Zacherl^62^ | 2000 | NS | 1991-1998 | Austria | 7 | NS | NS | Median 62.1 | Median 13.6 | NS | Median 37,2 |

**Legend**: When not specified otherwise, means are presented with standard deviations (±) or 95%-CI. Medians are presented with IQR. **Abbreviations**: N_p_: number of patients. PLD: polycystic liver disease. PCS: prospective cohort study. RAPCD: retrospective analysis of prospectively collected data. RCS: retrospective cohort study. NS: no statement.

**Supplementary File 4B: Individual study results (outcomes)**

| **Study (subgroups)** | **Recurrence**  **(N_p_)** | **Relief**  **(N_p_)** | **Re-intervention**  **(N_p_)** | **Complications**  **(N_p_)** | **Conversion**  **(N_p_)** | **Mortality**  **(N_p_)** | **Operative time**  **(minutes)** | **Hospital stay**  **(days)** |
| --- | --- | --- | --- | --- | --- | --- | --- | --- |
| Ammori^1^ | 0 | 2 | 0 | 1 | NS | 0 | NS | NS |
| Andriani^2^ | 1 | NS | 1 | 2 | 0 | 0 | NS | Mean 2 ( 1-5) |
| Ardito^3^ | 2 | 47 | 2 | 0 | NS | 0 | Median 164 (50-240) | Median 5,2 (2-12) |
| Bai^4^ | 2 | NS | NS | 5 | NS | 0 | NS | Mean 4 |
| Caetano^5^ | 0 | NS | 0 | 3 | 1 | 0 | Mean 105 (60-180) | Median 4.1 (3-8) |
| Cappellani^6^ | 0 | NS | 0 | 0 | 0 | 0 | NS | NS |
| Debs^7^ | 6 | NS | 6 | 5 | 2 | NS | NS | NS |
| Descottes^8^ | 5 | NS | 5 | 0 | 1 | 0 | NS | NS |
| De Reuver^9^ | 6 | 20 | 5 | 1 | 0 | 0 | NS | Mean 7,5 (1-12) |
| Diez^10^ | 0 | 10 | 1 | 0 | NS | 0 | NS | Mean 4 (3-6) |
| Emmermann^11^ | 1 | 16 | 1 | 2 | 1 | 0 | Mean 100 (50-150) | Mean 5 (2-14) |
| Fabiani^12^ | 1 | NS | NS | 3 | 2 | 0 | Mean 82 (15-210) | Mean 5,3 (2-12) |
| Faulds^13^ | 0 | 5 | 0 | 1 | 0 | 0 | NS | Mean 1,2 (0-4) |
| Fiamingo^14^ | 2 | 15 | NS | 4 | 1 | 0 | Median 80 (45-120) | Median 8 (6-12) |
| Gall (PLD)^15^ | 11 | NS | 6 | 4 | NS | 0 | Median 105 (40-180) | Median 4 |
| Gall (No PLD)^15^ | 14 | NS | 2 | 7 | NS | 0 | Median 75 (40-170) | Median 3 |
| Gamblin^16^ | 0 | 46 | 0 | 9 | 0 | 0 | Median 178 (54-380 | Median 2 (1-11) |
| Gigot^17^ | 1 | 10 | 1 | 4 | 4 | 0 | NS | Mean 6,1 (3-17) |
| Gocho^18^ | 0 | NS | 0 | 0 | 0 | 0 | Median 144 (100-210) | 3 |
| Hansen^19^ | 3 | 16 | 1 | 3 | NS | 0 | Mean 150 (90-253) | Mean 2,5 (1-8) |
| Hansman^20^ | 0 | NS | 0 | 0 | NS | 0 | NS | NS |
| Heintz^21^ | 0 | 3 | 0 | 0 | 0 | 0 | Mean 68,33 (55-90) | Mean 5,33 (5-6) |
| Hsu^22^ | 0 | 5 | 0 | 0 | NS | 0 | Mean 148 ( 110-215) | Mean 4,3 (3-5) |
| Kabbej^23^ | 8 | 11 | 4 | 8 | 0 | 0 | Median 190 (95-330) | Median 11 (3-30) |
| Kamphues^24^ | 6 | NS | 2 | 0 | 0 | 0 | Median 94 (30-195) | Median 5 (2-8) |
| Katkhouda (PLD)^25^ | 0 | 8 | 1 | 3 | 1 | 0 | Median 141 (94-165) | Median 3 (2-7) |
| Katkhouda (No PLD)^25^ | 0 | 16 | 0 | 1 | 0 | 0 | Median 48 (45-56) | Median 1,3 (1-3) |
| Kisiel^26^ | 9 | 46 | 3 | 5 | 0 | 0 | NS | Median 2 (1-7) |
| Koea (PLD)^27^ | 2 | NS | 2 | 0 | 0 | 0 | Median 87 (63-130) | Median 2 (2-7) |
| Koea (No PLD)^27^ | 0 | 12 | 0 | 0 | 0 | 0 | Median 50 (27-67) | Median 1 |
| Konstadoulakis^28^ | 2 | 9 | 1 | 1 | 1 | 1 | Mean 119,6 (55-179) | Mean 4,6 (2-6) |
| Koperna^29^ | 1 | 9 | 1 | NS | 1 | 0 | NS | NS |
| Kornprat^30^ | 0 | NS | 0 | 3 | 1 | 0 | Mean 100 (70-120) | Mean 10 (8-14) |
| **Study (subgroups)** | **Recurrence**  **(N_p_)** | **Relief**  **(N_p_)** | **Re-intervention**  **(N_p_)** | **Complications**  **(N_p_)** | **Conversion**  **(N_p_)** | **Mortality**  **(N_p_)** | **Operative time**  **(minutes)** | **Hospital stay**  **(days)** |
| Kwon^31^ | 0 | 14 | 0 | 0 | 0 | 0 | Mean 93 (40-170) | Median 7,0 (6-10) |
| Lee^32^ | 1 | NS | 1 | ? | 0 | 0 | NS | NS |
| Lolle Noerregaard^33^ | 5 | 26 | 3 | 2 | 2 | 0 | NS | Median 1 (1-14) |
| Manterola^34^ | 0 | NS | 0 | 0 | 0 | 0 | Median 50 (35-90) | Median 1 (1) |
| Marks^35^ | 0 | NS | 0 | 6 | 1 | 0 | NS | NS |
| Martin (PLD)^36^ | 5 | NS | 5 | 2 out of 5 | NS | 0 | Median 120 (75-180) | Median 3 (1-7) |
| Martin (No PLD)^36^ | 2 | NS | 2 | 3 out of 5 | NS | 0 | Median 60 (45-155) | Median 3 (1-10) |
| Martinez-Perez^37^ | 2 | NS | 1 | 2 | 1 | 0 | Median 122 (60-210) * | Mean 3,7 (1-7) * |
| Maruyama^38^ | 3 | NS | 0 | 2 | NS | 0 | Mean 165,8 (90-270) | Mean 17,8 (10-38) |
| Mazoch^39^ | 0 | NS | 0 | 2 | NS | 0 | NS | Mean 5,57 |
| Mazza (PLD)^40^ | 1 | NS | 1 | 5 | NS | 0 | NS | Mean 3 (1-6) |
| Mazza (No PLD)^40^ | 1 | NS | 1 | 1 | NS | 0 | NS | Mean 1,47 (1-3) |
| Morino (PLD)^41^ | 2 | 5 | NS | 4 | 2 | 0 | Mean 202 (149-295) | Mean 13 (4-25) |
| Morino (No PLD)^41^ | 0 | 4 | 0 | 0 | 0 | 0 | Mean 80 (40-135) | Mean 4 (2-6) |
| Neri (PLD)^42^ | 0 | 3 | 0 | 3 | 0 | 0 | Mean 120 (80-150) | Mean 6 (4-14) |
| Neri (No PLD)^42^ | 0 | 12 | 0 | 2 | 0 | 0 | Mean 55 (40-90) | Mean 6 (4-14) |
| Palanivelu^43^ | 0 | NS | 0 | 3 | 0 | 0 | Mean 72 (55-104) | Median 4 |
| Pante^44^ | 2 | NS | NS | 4 | 0 | 0 | Mean 98 (75-130) | Mean 6 (3-13) |
| Petri^45^ | 7 | NS | NS | 3 | NS | 0 | NS | 7 |
| Regev^46^ | 1 | NS | 1 | 0 | 0 | 0 | NS | NS |
| Robinson^47^ | 8 | 11 | 8 | 3 | 1 | 0 | Mean 75 ± 17 | Median 1 (0-7) |
| Roesch Dietlen^48^ | 0 | 7 | 0 | 1 | 0 | 0 | Mean 72 (40-110) | Mean 2 (1-3) |
| Sasi Szabo^49^ | 1 | NS | 1 | 2 | 2 | 0 | NS | Mean 4,7 (3-37) |
| Schachter^50^ | 1 | NS | 1 | 0 | 1 | 0 | NS | Mean 5,8 (3-10) |
| Scheuerlein^51^ | 0 | NS | 0 | 6 | 2 | 0 | NS | Mean 4 (1-18) |
| Sendt^52^ | 1 | NS | 1 | 0 | 0 | 0 | Mean 182 (72-270) | Mean 9,4 (7-12) |
| Tagaya^53^ | 1 | NS | 1 | 0 | NS | 0 | Mean 70 (60-225) | Mean/Median 6 |
| Tan^54^ | 2 | NS | 2 | NS | NS | NS | Mean 78 ± 20 | Mean 6,0 ± 5,0 |
| Tocchi^55^ | 0 | 20 | 0 | 0 | NS | 0 | NS | Mean 2-3 |
| Torices^56^ | 0 | NS | 1 | 2 | 0 | 0 | Mean 85 (53-110) | Mean 3,5 (2-9) |
| Torres^57^ | 2 | 31 | 1 | 6 | 3 | 0 | Median 85 (30-145) | Median 5 (2-29) |
| Treckmann^58^ | 1 | NS | NS | 3 | NS | 0 | Median 123,5 (50-318) | Median 3,5 (1-8) |
| Van Keimpema^59^ | 1 | NS | 1 | 1 | 1 | 0 | NS | NS |
| Wahba^60^ | 1 | NS | NS | NS | 0 | 0 | 58,3 ± 7,43 | 4,8 ± 0,44 |
| Wu (SILS)^61^ | 0 | NS | 0 | NS | 0 | 0 | 58,7 ± 6,14 | 5,2 ± 0,56 |
| **Study (subgroups)** | **Recurrence**  **(N_p_)** | **Relief**  **(N_p_)** | **Re-intervention**  **(N_p_)** | **Complications**  **(N_p_)** | **Conversion**  **(N_p_)** | **Mortality**  **(N_p_)** | **Operative time**  **(minutes)** | **Hospital stay**  **(days)** |
| Wu (standard)^61^ | 1 | 7 | 1 | 1 | 0 | 0 | Median 87 (30-180) | NS |
| Zacherl^62^ | 1 | 7 | 1 | 1 | 0 | 0 | Median 87 (30-180) | NS |

**Legend**: When not specified otherwise, means are presented with standard deviations (±) or 95%-CI. Medians are presented with IQR. **Abbreviations**: N_p_: number of patients. NS: no statement.

**References:**

1. Ammori BJ, Jenkins BL, Lim PC, Prasad KR, Pollard SG, Lodge JP. Surgical strategy for cystic diseases of the liver in a western hepatobiliary center. *World J Surg.* 2002;26(4):462-469.

2. Andriani O, Grondona J, Secchi M, et al. Laparoscopic approach for the treatment of symptomatic non-parasitic liver cysts is effective and minimally invasive. *HPB.* 2000;2(2):83-86.

3. Ardito F, Bianco G, Vellone M, et al. Long-term outcome after laparoscopic fenestration of simple liver cysts. *Surgical Endoscopy and Other Interventional Techniques.* 2013;27(12):4670-4674.

4. Bai XL, Liang TB, Yu J, et al. Long-term results of laparoscopic fenestration for patients with congenital liver cysts. *Hepatobiliary and Pancreatic Diseases International.* 2007;6(6):600-603.

5. Caetano EM, Linhares MM, Matos D, Schraibman V, Matone J, Saad SS. Laparoscopic management of hepatic cysts. *Surgical Laparoscopy Endoscopy & Percutaneous Techniques.* 2006;16(2):68-72.

6. Cappellani A, Zanghi A, Di Vita M, Menzo EL, Conti P. Nonparasitic cysts of the liver: laparoscopic treatment and long-term results. *Annali Italiani Di Chirurgia.* 2002;73(1):85-88; discussion 89.

7. Debs T, Kassir R, Reccia I, et al. Technical challenges in treating recurrent non-parasitic hepatic cysts. *International Journal of Surgery.* 2016;25:44-48.

8. Descottes B, Lachachi E, Durand-Fontanier S, Sodji M, de Laclause BP, Valleix D. Laparoscopic management of solid and cystic liver tumours. Report of 33 cases. *Annales De Chirurgie.* 2000;125(10):941-947.

9. de Reuver P, van der Walt I, Albania M, Samra JS, Hugh TJ. Long-term outcomes and quality of life after surgical or conservative treatment of benign simple liver cysts. *Surgical Endoscopy and Other Interventional Techniques.* 2017:1-9.

10. Diez J, Decoud J, Gutierrez L, Suhl A, Merello J. Laparoscopic treatment of symptomatic cysts of the liver. *Br J Surg.* 1998;85(1):25-27.

11. Emmermann A, Zornig C, Lloyd DM, Peiper M, Bloechle C, Broelsch CE. Laparoscopic treatment of nonparasitic cysts of the liver with omental transposition flap. *Surg Endosc.* 1997;11(7):734-736.

12. Fabiani P, Iannelli A, Chevallier P, Benchimol D, Bourgeon A, Gugenheim J. Long-term outcome after laparoscopic fenestration of symptomatic simple cysts of the liver. *British Journal of Surgery.* 2005;92(5):596-597.

13. Faulds JM, Scudamore CH. Technical report of a novel surgical technique: Laparoscopic cyst fenestration and falciform ligament pedicle graft for treatment of symptomatic simple hepatic cysts. *Journal of Laparoendoscopic and Advanced Surgical Techniques.* 2010;20(10):857-861.

14. Fiamingo P, Tedeschi U, Veroux M, et al. Laparoscopic treatment of simple hepatic cysts and polycystic liver disease. *Surgical Endoscopy and Other Interventional Techniques.* 2003;17(4):623-626.

15. Gall TM, Oniscu GC, Madhavan K, Parks RW, Garden OJ. Surgical management and longterm follow-up of non-parasitic hepatic cysts. *HPB (Oxford).* 2009;11(3):235-241.

16. Gamblin TC, Holloway SE, Heckman JT, Geller DA. Laparoscopic Resection of Benign Hepatic Cysts: A New Standard. *J Am Coll Surg.* 2008;207(5):731-736.

17. Gigot JF, Metairie S, Etienne J, et al. The surgical management of congenital liver cysts: The need for a tailored approach with appropriate patient selection and proper surgical technique. *Surg Endosc.* 2001;15(4):357-363.

18. Gocho T, Misawa T, Suzuki F, et al. Single-incision laparoscopic surgery for giant hepatic cyst. *Asian Journal of Endoscopic Surgery.* 2013;6(3):237-240.

19. Hansen P, Bhoyrul S, Legha P, Wetter A, Way LW. Laparoscopic Treatment of Liver Cysts. *J Gastrointest Surg.* 1997;1(1):53-60.

20. Hansman MF, Ryan JA, Holmes JH, et al. Management and long-term follow-up of hepatic cysts. *American Journal of Surgery.* 2001;181(5):404-410.

21. Heintz A, Junginger T. Laparoscopic surgery of cysts of the liver, spleen and mesentery. German. *Deutsche Medizinische Wochenschrift.* 1995;120(7):201-204.

22. Hsu KL, Chou FF, Ko SF, Huang CC. Laparoscopic fenestration of symptomatic liver cysts. *Surgical Laparoscopy, Endoscopy and Percutaneous Techniques.* 2005;15(2):66-69.

23. Kabbej M, Sauvanet A, Chauveau D, Farges O, Belghiti J. Laparoscopic fenestration in polycystic liver disease. *Br J Surg.* 1996;83(12):1697-1701.

24. Kamphues C, Rather M, Engel S, Schmidt SC, Neuhaus P, Seehofer D. Laparoscopic fenestration of non-parasitic liver cysts and health-related quality of life assessment. *Updates Surg.* 2011;63(4):243-247.

25. Katkhouda N, Mavor E, Gugenheim J, Mouiel J. Laparoscopic management of benign cystic lesions of the liver. *Journal of Hepato-Biliary-Pancreatic Surgery.* 2000;7(2):212-217.

26. Kisiel A, Vass DG, Navarro A, et al. Long-term Patient-reported Outcomes After Laparoscopic Fenestration of Symptomatic Liver Cysts. *Surg Laparosc Endosc Percutan Tech.* 2017.

27. Koea JB. Cystic lesions of the liver: 6 years of surgical management in New Zealand. *New Zealand Medical Journal.* 2008;121(1277):61-69.

28. Konstadoulakis MM, Gomatos IP, Albanopoulos K, Alexakis N, Leandros E. Laparoscopic fenestration for the treatment of patients with severe adult polycystic liver disease. *Am J Surg.* 2005;189(1):71-75.

29. Koperna T, Vogl S, Satzinger U, Schulz F. Nonparasitic cysts of the liver: Results and options of surgical treatment. *World J Surg.* 1997;21(8):850-855.

30. Kornprat P, Cerwenka H, Bacher H, et al. Minimally invasive management of dysontogenetic hepatic cysts. *Langenbeck's Archives of Surgery.* 2004;389(4):289-292.

31. Kwon AH, Matsui Y, Inui H, Imamura A, Kamiyama Y. Laparoscopic treatment using an argon beam coagulator for nonparasitic liver cysts. *Am J Surg.* 2003;185(3):273-277.

32. Lee DH, Cho JY, Han HS, et al. Laparoscopic treatment of hepatic cysts located in the posterosuperior segments of the liver. *Annals of Surgical Treatment and Research.* 2014;86(5):232-236.

33. Lolle Noerregaard C, Patrick Ainswort A. Good results after laparoscopic marsupialisation of simple liver cysts. *Danish Medical Journal.* 2014;61 (6) (no pagination)(A4866).

34. Manterola C, Otzen T. Laparoscopic Surgery in Nonparasitic Cysts of the Liver: Results Observed in a Series of Consecutive Cases. *Surgical Laparoscopy, Endoscopy and Percutaneous Techniques.* 2016;26(4):308-312.

35. Marks J, Mouiel J, Katkhouda N, Gugenheim J, Fabiani P. Laparoscopic liver surgery. A report on 28 patients. *Surgical Endoscopy.* 1998;12(4):331-334.

36. Martin IJ, McKinley AJ, Currie EJ, Holmes P, Garden OJ. Tailoring the management of nonparasitic liver cysts. *Annals of Surgery.* 1998;228(2):167-172.

37. Martinez-Perez A, Alberola-Soler A, Domingo-Del Pozo C, Pemartin-Comella B, Martinez-Lopez E, Vazquez-Tarragon A. Laparoscopic surgery and polycystic liver disease: Clinicopathological features and new trends in management. *JMAS.* 2016;12(3):265-270.

38. Maruyama Y, Okuda K, Ogata T, et al. Perioperative Challenges and Surgical Treatment of Large Simple, and Infectious Liver Cyst - A 12-Year Experience. *PLoS One.* 2013;8 (10) (no pagination)(e76537).

39. Mazoch MJ, Dabbous H, Shokouh-Amiri H, Zibari GB. Management of giant liver cysts. *Journal of Surgical Research.* 2011;167(2):e125-e130.

40. Mazza OM, Fernandez DL, Pekolj J, et al. Management of Nonparasitic Hepatic Cysts. *Journal of the American College of Surgeons.* 2009;209(6):733-739.

41. Morino M, De Giuli M, Festa V, Garrone C. Laparoscopic management of symptomatic nonparasitic cysts of the liver: Indications and results. *Ann Surg.* 1994;219(2):157-164.

42. Neri V, Ambrosi A, Fersini A, Valentino TP. Laparoscopic treatment of biliary hepatic cysts: Short- and medium-term results. *HPB.* 2006;8(4):306-310.

43. Palanivelu C, Jani K, Malladi V. Laparoscopic management of benign nonparasitic hepatic cysts: A prospective nonrandomized study. *South Med J.* 2006;99(10):1063-1067.

44. Pante S, Di Dio V, Putorti A, et al. Laparoscopic cyst fenestration in the treatment of polycystic liver disease. *Annali Italiani Di Chirurgia.* 2014;85(3):298-303.

45. Petri A, Hohn J, Makula E, et al. Experience with different methods of treatment of nonparasitic liver cysts. *Langenbeck's Arch Surg.* 2002;387(5-6):229-233.

46. Regev A, Reddy KR, Berho M, et al. Large cystic lesions of the liver in adults: A 15-year experience in a tertiary center. *Journal of the American College of Surgeons.* 2001;193(1):36-45.

47. Robinson TN, Stiegmann GV, Everson GT. Laparoscopic palliation of polycystic liver disease. *Surgical Endoscopy and Other Interventional Techniques.* 2005;19(1):130-132.

48. Roesch Dietlen F, Perez Morales A, Diaz Blanco F, Martinez Fernandez S. Laparascopic surgical treatment of non-parasitic hepatic cyst. Spanish. *Revista de Gastroenterologia de Mexico.* 1999;64(2):56-60.

49. Sasi Szabo L, Takacs I, Arkosy P, Sapy P, Szentkereszty Z. Laparoscopic treatment of nonparasitic hepatic cysts. *Surg Endosc.* 2006;20(4):595-597.

50. Schachter P, Sorin V, Avni Y, et al. The role of laparoscopic ultrasound in the minimally invasive management of symptomatic hepatic cysts. *Surgical Endoscopy.* 2001;15(4):364-367.

51. Scheuerlein H, Rauchfuss F, Franke J, et al. Clinical symptoms and sonographic follow-up after surgical treatment of nonparasitic liver cysts. *BMC Surgery.* 2013;13:42.

52. Sendt W, Weber T, Retschke S, Altendorf-Hofmann A. Symptomatic non-parasitic liver cysts: early and long-term results of surgical management. German. *Zentralblatt Fur Chirurgie.* 2009;134(2):149-154.

53. Tagaya N, Nemoto T, Kubota K. Long-term results of laparoscopic unroofing of symptomatic solitary nonparasitic hepatic cysts. *Surgical Laparoscopy, Endoscopy and Percutaneous Techniques.* 2003;13(2):76-79.

54. Tan YM, Chung A, Mack P, Chow P, Khin LW, Ooi LL. Role of fenestration and resection for symptomatic solitary liver cysts. *ANZ Journal of Surgery.* 2005;75(7):577-580.

55. Tocchi A, Mazzoni G, Costa G, et al. Symptomatic nonparasitic hepatic cysts: Options for and results of surgical management. *Archives of Surgery.* 2002;137(2):154-158.

56. Torices E DE, Méndez G, et al. Tratamiento laparoscopico de los quistes hepaticos simples. *Revista Mexicana de Cirugıa Endoscopica.* 2004;6(1):11-18.

57. Torres OJ, Farias AM, Costa MH, Matias MM, Moreira PC, Cordeiro GM. Laparoscopic treatment of liver cysts. *Rev Col Bras Cir.* 2009;36(6):493-497.

58. Treckmann JW, Paul A, Sgourakis G, Heuer M, Wandelt M, Sotiropoulos GC. Surgical treatment of nonparasitic cysts of the liver: open versus laparoscopic treatment. *Am J Surg.* 2010;199(6):776-781.

59. van Keimpema L, Ruurda JP, Ernst MF, van Geffen H, Drenth JPH. Laparoscopic fenestration of liver cysts in polycystic liver disease results in a median volume reduction of 12.5%. *Journal of Gastrointestinal Surgery.* 2008;12(3):477-482.

60. Wahba R, Kleinert R, Prenzel K, Bangard C, Holscher AH, Stippel DL. Laparoscopic deroofing of nonparasitic liver cysts with or without greater omentum flap. *SLEPT.* 2011;21(1):54-58.

61. Wu S, Li Y, Tian Y, Li M. Single-incision laparoscopic surgery versus standard laparoscopic surgery for unroofing of hepatic cysts. *JSLS : Journal of the Society of Laparoendoscopic Surgeons / Society of Laparoendoscopic Surgeons.* 2014;18(2):246-251.

62. Zacherl J, Scheuba C, Imhof M, Jakesz R, Fugger R. Long-term results after laparoscopic unroofing of solitary symptomatic congenital liver cysts. *Surgical Endoscopy.* 2000;14(1):59-62.
